# Rumination and postnatal depression: A systematic review and a cognitive model

**DOI:** 10.1016/j.brat.2016.05.003

**Published:** 2016-07

**Authors:** Hannah DeJong, Elaine Fox, Alan Stein

**Affiliations:** a Department of Psychiatry, University of Oxford, Warneford Hospital, Oxford, OX3 7JX, UK; b Department of Experimental Psychology, University of Oxford, 9 South Parks Road, Oxford, OX1 3UD, UK

**Keywords:** Postnatal depression, Rumination, Contingent responsiveness, Parenting, Cognitive control, Cognitive biases

## Abstract

Postnatal depression (PND) confers risk for a range of negative child developmental outcomes, at least in part through its impact on parenting behaviour. Whilst the behavioural effects of depression on parenting are well established, the cognitive mechanisms that may mediate this effect are less well understood. The current paper proposes that rumination may be a key cognitive mechanism through which parenting is affected in PND, and provides a systematic review of the existing literature on rumination in the context of perinatal depression. The review identifies ten relevant papers. Eight are questionnaire-based studies examining the role of rumination in predicting future depression and/or mother-infant relationship outcomes, such as bonding. Two are experimental studies examining the effects of induced rumination on parenting behaviours. The results of the review are discussed, and remaining questions highlighted. We then present a new theoretical model, developed specifically for the perinatal context, and informed by existing models of rumination and worry. Our cognitive model emphasises the relationship between rumination, cognitive biases and cognitive control, and the impact of these variables on infant cue processing and subsequent parenting responses. The model provides a potential framework for future work in this area, and to guide the development of treatment interventions.

## Highlights

• Postnatal depression affects parenting behaviour and subsequent child outcomes.
• There is a need to understand cognitive mechanisms that mediate this effect.
• Rumination, cognitive biases and deficits in cognitive control are implicated.
• These features affect processing of infant cues and so alter parenting behaviour.
• A cognitive model is proposed, to guide future work in this area.

Postnatal depression (PND) is a psychological disorder, typically defined as involving an episode of depression during the first post-natal year. Point prevalence during the first three months post-partum is around 13% (Howard et al., 2014). Whilst many women will recover within a few months, around 30% experience more persistent depressive symptoms and there is a high risk of further depressive episodes (Howard et al., 2014). PND is considered to be phenomenologically equivalent to major depressive disorder (MDD) at other times, and is highly similar in terms of symptoms, course and prognosis. Some studies have suggested that somatic and sleep-related symptoms may be somewhat more prevalent in PND (e.g. Williamson, O'Hara, Stuart, Hart, & Watson, 2015), leading to the development of perinatal assessment measures that exclude these features. However, in general these disorders are understood to be similar in symptomology and presentation. PND differs from MDD primarily in the *timing* of the depressive episode, and consequently its *implications*. PND has onset either during pregnancy or in the first year postnatally, and thus has specific impacts due to the importance of this developmental window for the infant, and also due to the uniqueness of the mother-infant relationship. During this period the infant is developing rapidly, and is highly dependent on the parent.^1^

Post-natal depression represents an important target for support and intervention, not only because of the impact on maternal mental health but because PND also carries long-term risks for offspring. A range of negative child outcomes associated with PND have been reported across cognitive, behavioural and emotional domains (Stein et al., 2014). One potential mechanism underlying the negative impact on child outcomes is difficulties with parenting quality. Aspects of parenting quality that have been identified include reduced sensitivity and responsiveness to infant cues, as well as a tendency to interact in a way that is either excessively withdrawn or intrusive (Murray, Halligan, & Cooper, 2010). These parenting behaviours then confer risk for a range of negative child outcomes. Negative outcomes for the child seem to be particularly pronounced where maternal disorder is persistent or recurrent (Stein et al., 2014).

It is recognised that early environmental and social input is vitally important for healthy development in infants, and that the mother-infant relationship provides a key part of this input. Whilst depression is understood generally to have negative impacts on relationships and social functioning, which apply to the mother-infant relationship, there are also distinctive features. For example, social cues from the infant may be more ambiguous than from adult interactive partners; infants are extremely sensitive to the qualities of interaction and contingency of responding (e.g. Bigelow and Rochat, 2006, Nadel et al., 2001, Striano et al., 2006); infants rely on a close degree of contingency in order to make sense of the interaction, due to their limited ability to detect contingency over temporal delays (Bornstein and Manian, 2013, Keller et al., 1999). The mother-infant relationship is also unusual in its intensity and the amount of time devoted to this dyadic interaction. The implications of depression during the perinatal period are therefore somewhat unique, in terms of impact on parenting, the mother-infant relationship and on various child development outcomes.

PND then is understood to exert effects on the child, at least in part, via its behavioural impact on parenting. However, the cognitive features of PND that are likely to mediate this relationship are not well understood. This paper seeks to outline the existing literature on one specific cognitive feature of PND, that is, rumination. Recent developments have highlighted the important role played by rumination in the onset and maintenance of depression. These findings are beginning to influence treatment approaches within the adult mental health field, but have not yet been broadly applied in the perinatal context. We suggest that integrating these developments into understanding PND provides helpful clues as to the mechanisms through which maternal depression may impact on child development.

Rumination is a key cognitive feature of depression and is defined as ‘a mode of responding to distress that involves repetitively and passively focusing on the symptoms of distress and the possible causes and consequences of these symptoms' (Nolen-Hoeksema, Wisco, & Lyubomirsky, 2008). Loss is hypothesised to be the core theme of rumination, and it is characterised by sustained and repetitive processing of negative emotional material. People who frequently ruminate when distressed are more likely to develop depression and are likely to remain depressed for longer. Rumination therefore confers risk for prolonged episodes and for future depressive episodes. The tendency to ruminate is trait-like and appears to remain fairly stable, even as depression improves. It has also been suggested that rumination is a trait vulnerability factor, as elevated rumination is found in never-depressed offspring of depressed parents (Nolen-Hoeksema et al., 2008).

Several authors have suggested that rumination may be an important cognitive mechanism through which perinatal psychological disorders affect parenting (Psychogiou and Parry, 2014, Stein et al., 2009). Stein and colleagues in particular have hypothesised that rumination is a form of preoccupation, which affects mothers’ ability to effectively process infant cues and results in reduced contingency and sensitivity in parenting behaviour. Whilst rumination has received considerable attention as a key cognitive feature in depression, it has been much less extensively characterised in the context of PND. In this paper, we seek to provide the first systematic review of evidence relating to rumination in the perinatal context. Drawing on this literature, as well as broader understandings of rumination, we then present a new framework for conceptualising the specific impact of rumination in the postnatal context. It is intended that this model can be used to generate hypotheses and to guide future research in this area.

We focus specifically on rumination because of its recognised importance in depression, and because theoretical accounts propose that this may be a key process in explaining the impact of PND on parenting. This is not however intended to imply that this is the only area of cognition or emotional regulation that is important within PND. Similarly, whilst we focus here on PND, there are likely to be parallels with other perinatal psychological disorders, e.g. anxiety disorders, eating disorders. Stein et al. (2009) argue that all these disorders are characterised by preoccupation, whether in the form of rumination, worry or obsessions, and that the mechanisms through which parenting is affected may then be very similar across disorders. Similar cognitive processes may therefore have broad applicability across a range of perinatal mental health conditions, but here we focus particularly on rumination in the context of depression.

## 1 Method

A systematic review of the literature was conducted, following the PRISMA guidelines (Moher, Liberati, Tetzlaff, & Altman, 2009). The search was conducted using several online databases: PsychInfo, Pubmed, MEDLINE and Scopus. The most recent search was completed in March 2016. Search terms included “*perinatal depression” OR “postnatal depression” OR “postpartum depression” OR “maternal depression”*, in combination with “*rumination” OR “repetitive negative thought” OR “repetitive negative thinking” OR “perseverative thought”*. Papers identified in this search were also used to check for additional results, by examining references and citations. In order to be included in the review, papers had to report data on some measure of rumination, or to report on the effects of a rumination induction. Results were limited to the perinatal period (i.e. measured during pregnancy or the first year postnatally). Review papers and opinion pieces were not included in the systematic review. Inclusion criteria also included publication in a peer reviewed journal, and availability in the English language.

**Fig. 1:**
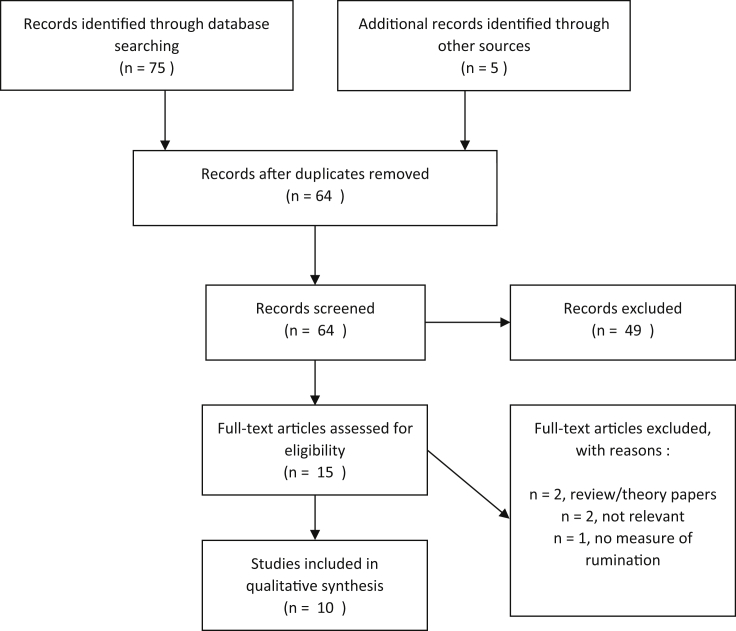
PRISMA diagram displaying search and screening process.

Search results were combined and duplicates removed, before titles and abstracts were screened. Full texts were then screened for relevance and fulfilment of the inclusion criteria. The search and screening process is shown in Fig. 1. There were only a small number of papers identified, with varied questions addressed and a wide range of methods and measures used. A qualitative synthesis or meta-analysis of the data is therefore not feasible or appropriate. Instead, a narrative synthesis of the findings is presented below.

## 2 Results

Ten papers were identified, addressing a range of questions about rumination within the perinatal context. Eight of these papers examined whether rumination is predictive of other maternal outcomes, including mood/depression and bonding with the infant, using questionnaire measures. Two papers described experimental studies examining the effects of induced rumination on some aspect of parenting behaviour. Each paper is described briefly below and study details are presented in Table 1.

### 2.1 Questionnaire studies

Five papers using questionnaire measures examined whether rumination is predictive of depression in the perinatal period, either alone or in combination with other variables. Barnum et al. (2013) showed that antenatal rumination, measured in the third trimester, was predictive of increased depressive symptoms at 8 weeks postpartum, although not at 4 weeks postpartum. Attributional style was also a predictor of later depressive symptoms, although only in women with pre-existing high levels of depression, suggesting it may be a maintenance factor. In contrast, Raes et al. (2014) found that rumination in the third trimester did not predict depression at 12 or 24 weeks, when controlling for previous episodes of depression. This may be because previous depressive episodes were very highly correlated with rumination. Depression during pregnancy and previous depressive episodes were key predictors of postnatal depressive symptoms, as was dampening of positive emotion.

O'Mahen et al. (2010) found a more complex relationship during pregnancy, such that rumination was predictive of later depression scores but only for women who reported poor social functioning. This suggests that effective social support may buffer the impact of rumination on future depressed mood. In contrast ‘silencing the self’ was predictive of rumination and depressed mood in women high in social functioning, but not those low in social functioning. These findings suggest that rumination may have a rather complex predictive relationship with perinatal depression, interacting with other risk and protective variables, both in the woman and in her social/environmental context.

O'Mahen, Karl, Moberly & Fedock (2015) examined links between childhood maltreatment, emotional regulation and depression in a group of women assessed during pregnancy. These women were either depressed or had elevated (but non-clinical) symptoms of depression. The data showed that brooding rumination was associated with higher levels of childhood maltreatment and with higher levels of depressive symptoms. Modelling showed that brooding rumination was a partial mediator of the relationship between childhood emotional abuse and current depressive symptoms. Brooding is a component of rumination that involves making passive comparisons between one's current situation and some unachieved standard, and is considered to be a particularly maladaptive subtype of rumination. This is contrasted with reflective rumination, which involves a more purposeful focus on difficulties in order to facilitate problem solving (Treynor, Gonzalez, & Nolen-Hoeksema, 2003). These findings therefore suggest that rumination, particularly the brooding form, may be a pathway through which childhood maltreatment increases risk of later depression. The authors hypothesise that this effect may be mediated through attachment style – emotional abuse has been shown to be linked to the development of anxious attachment, which in turn predicts higher levels of rumination. Thus they suggest that attachment styles emerge from experiences of abuse or maltreatment and lead to distinctive emotional regulation strategies, including rumination, which then confer increased risk of depression.

Finally, one study examined positive beliefs about rumination, comparing these across a depressed and non-depressed pregnant sample. Beliefs about the utility of rumination, for example as a strategy for problem solving or resolving unmet goals, are thought to be important as they may promote engagement in rumination (Papageorgiou and Wells, 2001, Papageorgiou and Wells, 2003). Isa Alfaraj et al. (2009) showed that positive beliefs about rumination were higher in a depressed than a non-depressed group, and that these beliefs predicted depression classification. This predictive relationship existed over and above perceived lack of social support.

The remaining three questionnaire-based studies examined impact of rumination on later bonding, attachment or self-reported maternal responsiveness. Müller et al. (2013) reported that rumination during pregnancy predicted mother-reported difficulties in bonding and attachment, even when controlling for pre- and post-natal depressive symptoms. The ‘unproductiveness’ subscale of the Perseverative Thinking Questionnaire (PTQ; Ehring et al., 2011) was particularly predictive of mother-infant bonding and attachment. This subscale includes items such as ‘my thoughts take up all my attention’ and ‘I keep asking myself questions without finding an answer’. These findings are consistent with the proposed role for rumination in capturing attention and impeding effective engagement with infant cues, resulting in interactive difficulties. However, as rating of the mother-infant relationship was self-reported, it is also possible that the effect of rumination is to impact maternal *perceptions* of bonding and attachment. Scores on the PTQ did not predict postnatal depressive symptoms.

In a recent study, Schmidt et al. (2016) examined changes in rumination, worry, depression, anxiety and maternal-foetal bonding from the first to third trimester of pregnancy. Maternal-foetal bonding is considered an important outcome because it is predictive of later mother-infant attachment and maternal sensitivity (Shin et al., 2006, Siddiqui and Hägglöf, 2000). Schmidt and colleagues showed that rumination in the first trimester had a small negative effect on intensity of maternal preoccupation with the foetus in the third trimester. Rumination was not predictive of later depression or anxiety symptoms, when controlling for baseline depression and anxiety. However, worry did have a small predictive effect for both depression and anxiety.

The role of rumination may also differ depending on infant factors. The final questionnaire study identified in the review indicated that rumination mediated the relationship between maternal depression and self-reported responsiveness to the infant, but only where infants displayed low levels of negative affect. Where infants had high levels of negative affect, depression was instead directly related to responsiveness (Tester-Jones et al., 2015). This finding was based on self-report measures and therefore it is unclear whether these relationships would also hold for observed maternal responsiveness. It does however highlight the possible impact of infant variables, and the complex interplay of these factors with maternal mood and cognitions. It is unclear why rumination appears to be implicated only for infants high in negative affect. However, the authors speculate that rumination may be more prevalent under conditions where mood is low in the absence of infant difficulties. Rumination is often used as a strategy to gain insight or resolve ambiguity, and therefore may be utilised in this causally ambiguous situation, i.e. where the parent is unhappy and finding parenting difficult but does not perceive the infant to be difficult or high in negative affect.

### 2.2 Experimental studies

Two experimental studies were identified – in both cases a rumination induction procedure was used before examining performance on some measure of parenting ability. Stein et al. (2012) tested whether inducing rumination or worry affected maternal responsiveness to 10 month old infants. This question was explored in a large sample composed of healthy controls and women meeting criteria for Generalised Anxiety Disorder (GAD) or MDD. Mother-infant interactions were examined both before and after a priming condition – either a worry/rumination prime, or a neutral prime. Worry/rumination was readily induced, using a simple procedure in which mothers were asked to spend 5 min thinking about either a current worry or a negative life event. Mothers in this condition reported more negative thoughts, more repetitive thoughts and more difficulty controlling thoughts, compared to the neutral prime condition; they also reported more negative affect. The impact of the priming was particularly strong for the clinical groups (GAD, MDD). This suggests that ruminative thinking styles were readily accessible and easily triggered in these groups, using a brief, simple priming procedure.

We focus here particularly on findings for the MDD group, although the full analysis included comparisons between GAD, MDD and controls. At baseline, the only difference between groups was that mothers with MDD followed their children's attention less than controls. Following the rumination prime, responsiveness to infant vocalization was lower in MDD than controls; this was not the case for the neutral prime. Also, across the MDD group, maternal responsiveness to infant vocalization was lower following the rumination prime than following the neutral prime. These were largely trend-level effects, with somewhat stronger effects found for the GAD group. None of these effects was moderated by remission status, suggesting that both recently recovered and currently anxious/depressed mothers responded similarly to the priming, and differed significantly from the control group. These findings therefore suggest that maternal cognitions, including rumination, have causal, observable effects on mother-infant interactions in the context of postnatal depression and anxiety. It is interesting to note that the main effects of the worry/rumination prime were on maternal responsiveness to *vocalization* specifically. It has been suggested that worry and rumination are primarily verbal in modality (Ehring & Watkins, 2008), and therefore may particularly impact on processing of concurrent auditory/verbal stimuli.

In the final study identified, O'Mahen et al. (2015) examined the impact of rumination on parental problem solving. Rumination in general is known to affect problem solving and is associated with difficulties both in generating solutions and then implementing these (Nolen-Hoeksema et al., 2008). Dysphoric and non-dysphoric women with an infant under 12 months were randomised to either a rumination or distraction condition. Problem solving effectiveness was then assessed using a parenting-specific problem solving measure, which involved generating solutions for problem scenarios in order to reach a specified outcome. (e.g. *Problem* – New to area, attend baby-mum class, baby cries uncontrollably, 2-year old bites another child. *Outcome* – I make some friends.) The results showed that, compared to all other groups, dysphoric mothers in the rumination condition demonstrated less effective problem solving and also rated themselves as less confident in their problem solving. The authors of this paper hypothesise that rumination captures attention and cognitive resources, and so impedes the ability to notice and engage with key features of the environment, resulting in lack of accurate information on which to base problem-solving strategies.

## 3 Discussion

The review highlights the rather mixed evidence as to whether rumination predicts later depression in the perinatal context, particularly after controlling for the effects of previous and current depression (e.g. Raes et al., 2014). Given the strong correlations of rumination with both current depression and history of depression, this may partly be because small studies have limited power to test predictive models and disentangle these effects. However, several papers indicate that rumination may interact with other variables including mother, infant and social/environmental factors (O'Mahen et al., 2010, O'Mahen et al., 2015), suggesting that rumination has a complex role in predicting future depression, which depends on the presence of other factors. The review also indicates that the time course over which rumination is examined may play a role, with for example rumination predicting later (2 months postnatal) but not earlier (1 month postnatal) depression (Barnum et al., 2013). Positive beliefs about the utility of rumination may also be important (Isa Alfaraj et al., 2009), although this was examined only concurrently with mood, and not in a longitudinal design. Very few perinatal studies have distinguished between the different components of rumination, but where this was addressed, brooding rumination was particularly associated with depression and also mediated the relationship between childhood adversity and depression (Barnum et al., 2013, Tester-Jones et al., 2015). This is consistent with the suggestion that brooding is a particularly maladaptive form of rumination (Nolen-Hoeksema et al., 2008), and suggests that this may similarly be true in the perinatal context. Further research using measures that distinguish between brooding and reflective components of rumination would help to further explore this possibility.

The current review also indicates that rumination has an impact on relational outcomes such as bonding and attachment, with higher rumination predicting later difficulties with bonding and attachment (Müller et al., 2013, Schmidt et al., 2016, Tester-Jones et al., 2015). However, there are very few studies examining this question and the existing studies are longitudinal but do not allow definite causal conclusions to be drawn. Critically, these studies have considered only mother-reported indices of these outcomes, rather than objective measures. The use of objective measures seems particularly important given the likely role of low mood and rumination on *perception* of these outcomes. As discussed in relation to prediction of later depression, rumination is likely to interact with other factors such as social support and infant temperament to predict relational outcomes (Tester-Jones et al., 2015). Given these findings, it is important to extend this work by establishing under what circumstances, or in the context of what other variables, is rumination predictive of later maternal outcomes (e.g. depression) and mother-infant outcomes (e.g. bonding). Distinguishing between subtypes of rumination may also be of importance here; the only study in the review to do so demonstrated that *brooding* specifically was a partial mediator of the relationship between depression and self-reported responsiveness to the infant (Tester-Jones et al., 2015).

The existing experimental evidence suggests that inducing rumination (or similar processes, such as worry) has an impact on maternal responsiveness (Stein et al., 2012) and effectiveness of infant-related problem solving (O'Mahen et al., 2015). Experimental designs provide a more robust test of causality and suggest that rumination may indeed have a causal, mechanistic role. However, there are many remaining questions about the pathways through which rumination operates on parenting responses, the specific changes it causes, and subsequent impact on infants; further experimental research is clearly required.

The current review highlights some surprising gaps in the existing research. For example, there is currently no research examining issues such as the prevalence, chronicity and content of rumination in the perinatal context. Some qualitative research into women's experiences of PND has indicated themes that resemble rumination, but these are not typically labelled as such and therefore were not included in the systematic review. For instance, qualitative themes identified include: “obsessive thoughts of being a bad mother”, “wondering what was happening to them”, and “loss of control over thoughts” (C. T. Beck, 1992). Qualitative studies also frequently identify themes of loss, and incongruity between expectations and realities of motherhood (C. T. Beck, 2002). These ideas closely match the primary content of ruminative thought, that is, themes of loss and discrepancy between current and desired states (Nolen-Hoeksema et al., 2008). However, there is little systematic evidence regarding themes of rumination in PND, and how closely these parallel the content of rumination in depressive disorder more broadly. There is similarly no published literature on the prevalence or frequency of ruminative thought in PND. Recently, experience sampling methodology has been used to examine rumination in daily life (Kirkanski, Thompson, Sorenson, Sherdell, & Gotlib, 2015), and this method may be useful for addressing some of these questions in the perinatal period.

This systematic review therefore provides some information about the potential role of rumination in predicting later outcomes, including maternal depression and mother-infant outcomes such as bonding. There is also some initial evidence that rumination plays a causal role in impairing parenting quality in clinical populations. However, the review also highlights many remaining questions and areas where this work could be extended. We have briefly outlined some possible future directions here and discuss this in more detail later in the paper (*see* Section 3.8).

To date, the literature on rumination in the perinatal context has lacked a theoretical model or organising framework. Therefore, for the remainder of this paper, we develop and present a new cognitive model which seeks to account for the specific effects of rumination in the postnatal context. This model is informed by the literature identified in the systematic review, particularly initial findings that inducing rumination has measurable effects on parenting quality. We have also built upon existing theories and models of rumination, taken from the broader depression literature, and so a brief overview of the frameworks and theories that have most influenced our model is presented below.

### 3.1 Theoretical models of rumination and related cognitive processes

Various information-processing models have been proposed that seek to explain how rumination is triggered and maintained in the context of depression. Many of these models emphasise the role of cognitive biases and cognitive control deficits (reviewed in Mor & Daches, 2015). These models of rumination tend to implicate either absolute deficits in cognitive control or biases in the deployment of cognitive control, such that processing of certain types of information (e.g. negative or self-referential information) is less effectively regulated. Cognitive control refers to a set of processes that facilitate flexible cognition and behaviour, in line with moment-to-moment goals. This concept is closely related to executive functions, and is generally held to include processes such as overriding pre-potent responses and inhibiting processing of irrelevant information, switching the focus of attention, and updating working memory (Miyake et al., 2000). Difficulties with inhibition of irrelevant information and flexibly shifting the focus of attention have been particularly implicated in rumination (Davis and Nolen-Hoeksema, 2000, De Lissnyder et al., 2011, Joormann, 2006, Whitmer and Banich, 2007). It is hypothesised that deficits or biases in these domains could account for difficulties in terminating ruminative thought processes once these are triggered. This may help to account for the prolonged and perseverative quality of rumination. There is then an emerging consensus that cognitive control difficulties, whether absolute deficits or biases, are implicated in rumination. It has been suggested that cognitive control deficits or biases may in fact play a causal role in rumination and depression. Evidence for this is starting to emerge from longitudinal studies (Demeyer, De Lissnyder, Koster, & De Raedt, 2012) and experimental designs (Mor & Daches, 2015). However, this relationship may be bi-directional, as there is evidence that inducing rumination impairs aspects of cognitive control (Philippot & Brutoux, 2008; E.; Watkins and Brown, 2002, Whitmer and Gotlib, 2013).

Other, related models conceptualise rumination as being akin to a cognitive habit, which develops in the context of deficits in cognitive control. This idea was proposed by Hertel (2004) and has been further developed in a recent model from Watkins and Nolen-Hoeksema (2014). Watkins & Nolen-Hoeksema suggest that habitual, trait rumination develops through the repeated co-occurrence of rumination with contextual cues (e.g. low mood), such that rumination eventually becomes automatically cued by these contextual factors. They argue that deficits in cognitive control may confer vulnerability to habitual rumination, as cognitive control enables people to override pre-potent or habitual responses, in order to respond flexibly to situational demands. Similarly, Segal and colleagues (e.g. Segal, Williams, & Teasdale, 2013) have proposed that during repeated episodes of depression, a strong connectivity develops between depressed mood and depressogenic cognition, including rumination. This connectivity creates a heightened cognitive vulnerability, meaning that when low mood is experienced in future this triggers high levels of depressogenic cognition, and so increases the risk of further episodes of depression.

Forms of repetitive negative thinking, such as rumination, are increasingly recognised as transdiagnostic phenomena (De Raedt, Hertel, & Watkins, 2015), with relevance to both anxiety and depressive disorder. Various accounts of both anxiety and worry have been proposed that have clear parallels with the models outlined above in terms of the emphasis on the role of cognitive biases and cognitive control deficits. Therefore, in addition to models from the depression literature, our cognitive model is also informed by existing models of anxiety and worry. We have particularly drawn on Attentional Control Theory (Eysenck and Derakshan, 2011, Eysenck et al., 2007) and a key model of worry (Hirsch & Mathews, 2012). These accounts are similar in that they integrate ‘bottom up’ biases in attentional processing with ‘top down’ cognitive control processes. They may therefore have utility in guiding the development of an integrative model of rumination, cognitive biases and cognitive control.

### 3.2 Cognitive biases in perinatal depression

Cognitive biases have been clearly implicated in the development and maintenance of rumination (Everaert et al., 2012, Mathews and MacLeod, 2005), but much less work has been conducted specifically in perinatal populations. In a recent review, Webb and Ayers (2014) identified 14 studies examining cognitive biases in women with perinatal mental health problems, including depression, anxiety and PTSD. Various study designs were employed, but in each case the stimuli examined were either adult or infant facial expressions. There is a particular interest in attention to and interpretation of emotional facial expressions in this population, as it has been argued that the ability to interpret infant emotion accurately is a prerequisite for sensitive parenting behaviour. Webb and Ayers concluded that women who are depressed tend to show attentional biases towards sad infant faces, and to a lesser extent show similar biases towards sad adult faces. There is also evidence that this group of mothers are more likely to interpret neutral or ambiguous infant faces as negative. These biases seem likely to negatively affect contingent, sensitive parenting because this relies on an ability to accurately detect infant cues, in order to respond in an appropriately matched way. If a mother is biased towards detection of negative cues, she may miss important positive or neutral cues, such as happiness or interest, which would otherwise have guided her responses. Infant cues also provide critical feedback that allows mothers to continually evaluate and adjust their parenting responses as required. Cognitive biases in infant cue perception may therefore affect mothers' evaluations of their parenting, and ability to adjust responses to meet the infant's needs.

The perinatal literature is therefore small but broadly consistent with the general depression literature in suggesting negative cognitive biases in attention and interpretation. Various questions have not yet been addressed, including whether memory biases are present and how these different cognitive biases relate to one another. Biases in attention, interpretation and memory are likely to interact with one another; for example an attentional bias towards negative information may lead to more negative information being recalled later (memory bias). Therefore, in interaction with one another, these biases may have wide-ranging impacts on perception of the infant (e.g. affect, temperament), the self (e.g. self-efficacy, evaluation of parenting ability), and the parent-infant relationship (e.g. bonding, attunement). It is also unclear from the current literature in PND whether cognitive biases operate on a range of stimuli, including facial expressions other than sadness (e.g. anger, fear) and vocal stimuli; this may be a useful direction for future research.

### 3.3 Cognitive control deficits in perinatal depression

As outlined above, cognitive control deficits are associated with rumination and considered to be important for understanding vulnerability to rumination. Impairments in cognitive control are perhaps particularly relevant for postnatal populations, given the demands that parenting places on executive function. Barrett and Fleming (2011) note that mothers “must be flexible and be able to focus when appropriate and switch attention when the situation changes … planning, attention, flexibility and working memory are essential for adequate mothering”. A handful of studies using neuropsychological measures have found evidence of impaired executive function associated with postnatal depression. For example, Pio de Almeida, Jansen, Köhler, Pinheiro, da Silva (2012) report evidence of impaired working and short-term memory in depressed parents; Messinis et al. (2010) report poorer performance on a learning task and an attention switching task by postnatally depressed women compared to controls. However, there has been little work in this area and a more thorough examination of cognitive control in PND is indicated. It would also be of interest to explore links between cognitive control in PND and changes in parenting behaviour, particularly the features of parenting that are critical to child development, such as contingency.

### 3.4 Applying a theoretical model to postnatal depression

There are then several theoretical models that seek to explain rumination, or related cognitive processes such as worry, with reference to underlying cognitive biases and/or cognitive control deficits. The literature in PND, reviewed above, is broadly consistent with findings from research in depression and rumination more broadly. However, the perinatal literature has not typically been guided by a specific theoretical framework and therefore there are many gaps in our knowledge. Use of such a framework may provide a focus for future research and advance understanding in this field.

Whilst the models of rumination and worry outlined above provide a helpful starting point for a theoretical framework, they require adaptation in order to account for particular effects in the context of PND. We propose an information-processing model of rumination in PND, guided by more general models of rumination and worry, which incorporates a focus on cognitive biases and cognitive control deficits, and the role of low mood/associated cues in triggering rumination. Importantly, our model also includes processes that are specific to the postnatal context, that is, effects on infant cue processing and parenting responses.

**Fig. 2:**
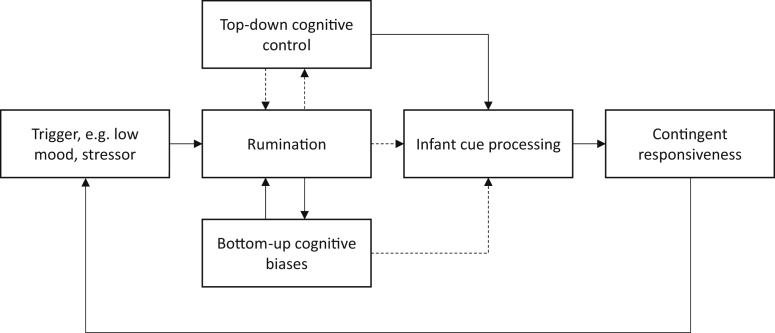
Information processing model of rumination and effects on parenting behaviour. Solid lines indicate positive links and dotted lines indicate negative/inhibitory links.

Our cognitive model of rumination in postnatal depression is intended to provide an initial theoretical framework that may illuminate some of the underlying cognitive mechanisms. These pathways provide testable hypotheses for future research and may also indicate possible intervention routes. Findings from attention bias modification and cognitive bias modification research suggest that cognitive biases and cognitive control deficits are potentially modifiable pathways, and therefore suitable targets for intervention. The model is described below and illustrated in Fig. 2. The description of the model begins with an account of rumination, and its relation to cognitive biases and cognitive control deficits, which is based on existing models from the broader depression literature. We then go on to discuss how these processes may specifically affect the postnatal period, with reference to their impact on infant cue processing and parenting behaviour.

### 3.5 A cognitive model of rumination and parenting

We propose that rumination is associated with both top-down cognitive control deficits and bottom-up cognitive biases. These components of the model are hypothesised to have bidirectional links with rumination. Within the model, rumination is preceded by an initial trigger, which may be internal, such as mood or an intrusive negative thought, or could be an external event such as an everyday stressor. In the postnatal context, this may include parenting related triggers, such as managing multiple demands or encountering infant negative affect. Following this trigger, rumination is established and maintained though the combined effects of cognitive biases and cognitive control deficits.

Firstly, it is suggested that in the context of depression or low mood, cognition is biased in favour of negative information. Negative information is therefore more likely to enter awareness and thus influences the content of ruminative thought, such that this is pervasively negative. Rumination, which involves repetition and elaboration of this content, likely also reinforces cognitive biases via rehearsal. These biases are therefore strengthened and it becomes increasingly likely that negative content and ruminative processes will be activated when future triggers are encountered.

Secondly, deficits in cognitive control are hypothesised to impair the ability to reappraise or redirect attention away from negative thoughts once triggered. Cognitive control incorporates voluntary, executive processes that facilitate directing attention towards task-relevant information in a goal-directed manner, by flexibly shifting the focus of attention and inhibiting distracting information. Typically then, cognitive control processes enable people to override negative, ruminative thought processes once triggered, and to redirect attention onto alternative topics or task-relevant information. However, in the context of cognitive control deficits, there may be insufficient control to override ruminative processes once these develop. These thoughts are therefore subject to continued attention and develop into a repetitive and persistent thought process. When this occurs persistently, a pattern of high trait rumination is established. Rumination is also likely to exert reciprocal effects on cognitive control, as it is an activity that consumes cognitive resources (Hayes et al., 2008, Stefanopoulou et al., 2014; Watkins & Brown, 2002). This may mean that during rumination, with cognitive control directed towards processing of the ruminative context, it becomes increasingly difficult to redirect attention. The ruminative state is therefore maintained and becomes prolonged.

The combined effects of negative cognitive biases and cognitive control deficits may therefore help explain vulnerability to rumination. This type of model is consistent with the characteristics of ruminative thought, particularly the reported experience that it is repetitive and uncontrollable. Within this model, rumination also acts to reinforce cognitive deficits and biases, thus increasing the likelihood that rumination will be triggered in future. This is consistent with the stability and persistence of trait rumination over time.

In the postnatal context, we propose that these cognitive processes will have important effects on infant cue processing. Cognitive biases are hypothesised to affect attention to and interpretation of infant emotional cues. Cognitive control deficits are hypothesised to affect the ability to appropriately direct cognitive resources towards infant cues. Biases and control deficits may therefore directly impair infant cue processing, potentially resulting in slower and/or less accurate perception of infant cues. In addition, it is proposed that cognitive biases and control deficits impair cue processing indirectly, via their actions on rumination. The combination of cognitive biases and control deficits leads to rumination becoming highly prevalent and persistent. It thus captures attentional and cognitive resources so that external cues, including infant cues, are likely to be less effectively or less rapidly attended. This is consistent with the proposed role of preoccupation as an important mechanism through which maternal mental health disorders affect parenting (Stein et al., 2009). Given the frequency and persistence of ruminative thinking in depression, it is hypothesised that its effects on parenting behaviour may be pervasive.

Failure to rapidly and accurately perceive infant cues is likely to have a significant impact on parent-infant interaction, particularly contingent responsiveness. Contingent responsiveness refers to a pattern of responding to the infant in which the parent's behaviours are closely matched to the infant's cues, both in terms of timing and selection of response (Bornstein & Tamis-LeMonda, 1989).

The mother's ability to deliver an appropriate and timely response, and so maintain contingency, is dependent on accurate and efficient processing of infant cues. We hypothesise that information processing deficits and biases, along with the preoccupation inherent in rumination, impair mothers' ability to process infant cues accurately and efficiently. Contingent responding is thus impaired. Infants are highly sensitive to deviations from contingency (Bigelow et al., 1996, Mesman et al., 2009, Tronick et al., 1978), and can perceive contingency only over short latencies (Keller et al., 1999). Therefore, even subtle mismatches or delays in responding may have significant implications for child outcomes.

Psychogiou and Parry (2014) have suggested that unstructured interactions (e.g. play) may be particularly affected as parental response is highly dependent on self-directed attention. In contrast, more structured situations may be less affected, as the externally imposed structure may help to focus and direct parental responses. Similarly, it has been shown that parenting difficulties associated with maternal psychopathology are mainly observed under conditions of stress or challenge (Ginsburg, Grover, Cord, & Ialongo, 2006). Within the model, this is consistent with the role of stress or challenge as a trigger for rumination, which then has effects on cue processing and interaction. Impairments in contingent responding are likely to impact on the mother's experience of parenting and sense of competence as a parent. Lack of contingency is likely to result in difficult or mismatched interactions with the infant, which may in fact act as a trigger to further rumination.

### 3.6 Implications for child development

Contingent responsiveness is thought to be a key aspect of effective parenting, which promotes the infant's development. If rumination, and related attentional processes, have effects on contingent responding therefore, this may be an important mechanism through which maternal mood impacts on child developmental outcomes. It has been suggested that contingent responsiveness is particularly implicated in child *cognitive* development. Contingent responsiveness in mothers with depression is predictive of children's attention and cognitive functioning (Murray et al., 2010), which in turn predicts later intellectual functioning (Bornstein, 2014, Stanley et al., 2004). It could therefore be hypothesised that high levels of maternal rumination might be particularly associated with risk of negative attentional and cognitive outcomes for the infant. PND in general is associated with a range of risks for child development, including behavioural and emotional, as well as cognitive outcomes (reviewed in Stein et al., 2014). It is possible that rumination, and subsequent changes in infant cue processing and contingency, play an important role in mediating some or many of these risks. There is a need for longitudinal, developmental studies to examine these questions.

Finally, whilst the research to date has focused on mothers, there is no reason to believe that similar processes might not also apply to fathers; this should be a focus of future work in this area.

### 3.7 Implications for intervention

The model presented here has implications for intervention design – it highlights specific cognitive features of PND that we argue are key mechanisms through which depression affects parenting and therefore child development. Interventions that target these cognitive mechanisms are therefore likely to have benefits both for maternal mental health, and for child outcomes.

Although rumination has rarely been examined as an outcome in treatment trials, there is some evidence that cognitive behavioural therapy does not effectively reduce rumination (Schmaling, Dimidjian, Katon, & Sullivan, 2002), that high levels of rumination predict poorer outcomes (Jones, Siegle, & Thase, 2008), and that rumination at end of treatment predicts depressive relapse (Michalak, Hölz, & Teismann, 2011). There is therefore increasing interest in treatments that are hypothesised to specifically target rumination, including mindfulness approaches (e.g. MBCT; Segal et al., 2013), acceptance-based approaches (Hayes, Strosahl, & Wilson, 2003), behavioural activation (Martell, Dimidjian, & Herman-Dunn, 2012) and metacognitive therapy (Wells, 2009). Given that residual rumination seems to confer risk of future depressive episodes, clinicians and researchers have suggested that more effectively targeting this aspect of depressive cognition may improve treatment outcomes and reduce relapse. In the perinatal context, one recent study examined the effectiveness of MBCT delivered during pregnancy, for women with a prior history of depression. It was found that, compared to treatment as usual, MBCT during pregnancy reduced postnatal depressive symptoms and postnatal risk of depressive relapse/reoccurrence (Dimijian et al., 2016). This suggests that preventative interventions targeting cognitive vulnerability factors may be beneficial, particularly for high risk groups.

In the postnatal context, treating depression alone does not seem to be sufficient to ameliorate negative effects on parent-infant interactions and child outcomes (Poobalan et al., 2007). One hypothesis is that residual rumination may be a factor underlying this effect; if ruminative thinking remains a habitual response that is triggered when low mood or stressors are encountered, then this would continue to effect parental attention to infant cues, and therefore negatively impact on parent-child interactions.

The model outlined above generates several testable predictions regarding the type of interventions that may be helpful. In particular, it suggests that interventions that shift the balance of competition between bottom-up biases and top-down control processes should be beneficial in reducing rumination. This could involve directly targeting ‘top-down’ attention control processes, ‘bottom-up’ attention biases, or some combination of both these factors. Interventions that target the ‘bottom-up’ cognitive biases in the model could include attention bias modification (ABM) procedures and cognitive bias modification procedures that target biases in interpretation of ambiguity (CBMi). These procedures are designed to change biases through repeated rehearsal of a more positive or neutral bias (Hertel & Mathews, 2011). ‘Top-down’ interventions may include cognitive remediation (Porter, Bowie, Jordan, & Malhi, 2013) or other training procedures, such as working memory training (e.g. Owens et al., 2014). There may be particular benefits to a ‘top-down’ approach as increased cognitive control is likely to facilitate greater *flexibility* of responding. This flexibility of responding has been proposed to be important for effective emotional regulation (Ochsner & Gross, 2005) and resilience (Genet & Siemer, 2011), and is likely to play a key role in facilitating sensitive and contingent parenting behaviour. The model presented here provides a framework to direct hypothesis generation and future systematic investigation of these questions.

### 3.8 Remaining questions and future directions

The review and theoretical framework presented here highlight many remaining questions about the role of rumination in PND, and suggest several potential avenues for future research. Firstly, there is a need for studies that better characterise rumination in the context of PND, including prevalence, frequency and persistence of rumination, as well as content and themes. More consistent use of terminology and better alignment with the general depression literature may be helpful in developing our understanding of the role of rumination in the postnatal context. Inclusion of rumination measures in treatment trials and epidemiological studies would also facilitate exploration of the role of rumination as a proposed vulnerability factor and mechanism of change.

Secondly, the proposed role of cognitive biases and cognitive control in the model could be tested directly. Experimental designs, which manipulate either cognitive biases or cognitive control, and examine impact on rumination, would provide a particularly robust test of the model. There are a small number of studies addressing these processes in non-perinatal groups, although to date findings have been mixed (Mor & Daches, 2015). As the literature on rumination and cognitive control in depression continues to develop, this may support or suggest refinements to the model.

Thirdly, the impact of rumination on infant cue perception and subsequent parenting behaviour requires further examination. Whilst there is evidence that rumination affects maternal responsiveness (Stein et al., 2012), the precise effects of rumination on infant cue processing are unclear. It may be that the effects of rumination differ according to the emotional tone of cues (e.g. happy, sad or neutral expressions), or that certain modalities (e.g. visual, auditory) are more affected than others. As rumination is thought to be predominantly verbal phenomena (Ehring & Watkins, 2008), it might be expected that responsiveness to auditory/vocal cues would be particularly affected. If rumination does indeed affect responsiveness to particular cues, this may be demonstrated by changes in either accuracy or reaction time, or both of these parameters. Experimental studies using standardised infant cue stimuli, in combination with induced rumination procedures, could be used to address these questions. In addition, examining infant cue perception and live mother-infant interaction in the same group would facilitate a test of the hypothesised links between these components in the model. Longitudinal designs would also be of use in extending this work to include direct measures of developmental outcomes for the infant.

## 4 Conclusions

There is a general need to better understand the mechanisms through which PND affects child outcomes. This is critical, in order to guide the design of more efficacious interventions that ameliorate these effects. The cognitive features of PND, including rumination and related cognitive deficits and biases, provide promising and relatively under-explored possible pathways. Increasing evidence from the broader treatment literature in depression suggests that these mechanisms are modifiable, either using psychotherapy or specific training interventions (e.g. ABM, working memory training). Interventions targeting these mechanisms therefore have potential to improve mother-infant interaction and child developmental outcomes. Given the role of rumination in conferring risk for future episodes of depression, interventions targeting this process may also have important benefits for long-term maternal mental health. In addition, it has been suggested that screening for and treating rumination may have potential as a preventative intervention (Topper, Emmelkamp, & Ehring, 2010). Increased contact with health services and motivation to make lifestyle changes during pregnancy and the postnatal period may make this a particularly suitable time for preventative work. Given the proposed similarities between rumination and other repetitive negative thought processes (e.g. worry, preoccupation), interventions targeting rumination may also have broader applicability, for example in anxiety disorders or other parental mental health conditions.

The theoretical framework outlined here may have utility in guiding future research and in the development of improved interventions for PND. It provides a theoretical account of potential maintenance mechanisms, and suggests several pathways that may be modifiable. Understanding of these mechanisms is likely to benefit from ongoing experimental and clinical studies of rumination more broadly. However, there is also a need to test these mechanisms directly in postnatal groups in order to examine the hypothesised effects on processing of infant cues and contingent responding, and ultimately on child developmental outcomes.

## Figures and Tables

**Table 1:** Summary of included studies.

Authors (year)	Participants	Measures / procedure	Findings
*Questionnaire studies*			
Barnum, Woody, and Gibb (2013)	N = 101 3rd trimester of pregnancy to 8 weeks postpartum. USA.	Rumination (RRS; 3rd trimester) Negative inferential styles (Expanded Attributional Style Questionnaire; 3rd trimester) Depression (EPDS; 3rd trimester, 4 and 8 weeks postpartum)	Brooding rumination and negative inferential styles predict depression at 8 weeks, but not 4 weeks. Attributional style only predictive for women with already high levels of depression – may be predictor of maintenance.
Isa Alfaraj, Spada, Nikčević, Puffett, and Meer (2009)	N = 21 depressed, N-22 non-depressed (assessed by BDI) 2nd or 3rd trimester of pregnancy. UK.	Positive beliefs about rumination (PBRS) Perceived lack of social support over previous month (rating scale).	Positive beliefs about rumination higher in depressed group. Perceived social support lower in depressed group. Positive beliefs about rumination predict depression classification above perceived lack of social support.
Müller, Teismann, Havemann, Michalak, Seehagen, (2013)	N = 66 2nd or 3rd trimester of pregnancy, up to 8 weeks postpartum. Germany.	Perseverative thinking (PTQ) Depression (BDI-II) Postpartum bonding (PBQ) Attachment Scale of PSI	PTQ does not predict postnatal depression. Unproductive subscale of PTQ predicts bonding and attachment (as does ante- and post-natal depression).
O'Mahen, Flynn, Nolen-Hoeksema (2010)	N = 110 scoring 10 or more on EPDS (N = 65 at follow up) During pregnancy and 3mths later. USA.	Assessment via phone interview, including: Mood (EPDS) Rumination (RRS; 10 item version) Social functioning (SASS) Silencing the self (STSS)	For low social functioning: rumination related to later depression; silencing the self unrelated to rumination or mood. For high social functioning: social functioning buffers impact of rumination on future depressed mood; silencing beliefs associated with increased rumination and depressed mood.
O'Mahen, Karl, Moberly, and Fedock (2015)	N = 55 depressed pregnant women, N = 85 elevated low mood (not clinically depressed) pregnant women. M = 23.27 weeks gestation. USA.	Mood (SCID-IV, EPDS, BDI-II). Childhood Trauma Questionnaire. Rumination (RRS). Behaviour activation (BADS).	Brooding rumination associated with depressed symptoms and also with history of neglect and abuse. Brooding partially mediated relationship between childhood emotional abuse and depression. Behaviour activation fully mediated relationship between childhood emotional neglect and depression. Brooding and behavioural activation inversely related.
Tester-Jones, O'Mahen, Watkins, Karl (2015)	N = 203 Infants 9–14 mths. UK.	Rumination (RRS) Depression (EPDS) Attunement and bonding (MIRI and PBQ) Perceived social support (SPS) Infant temperament (negative affect subscale of IBQ-R)	Brooding rumination mediated the effect of postnatal maternal depressive mood on maternal self-reported responsiveness to the infant when infants were low, but not high, in negative temperament. For infants with higher negative temperament, maternal depressive symptoms were directly related to self-reported responsiveness, as was perceived social support.
Raes et al. (2014)	N = 210 3rd trimester of pregnancy; follow up at 12 and 24 weeks postpartum. Belgium.	Depression (DASS; EPDS) History of depression (MDQ) Rumination (RRS) Dampening of positive emotions (RPA)	Depression during pregnancy and previous depressive episodes predict depression postpartum. Dampening also has predictive role, but rumination does not. Rumination highly related to previous depressive episodes. Similar findings at 12 and 24 months.
Schmidt, Seehagen, Vocks, Schneider, and Teismann (2016)	N = 215 Seen in 1st and 3rd trimester. Germany.	Rumination (RRS) Worry (PSWQ-PW) Depression and anxiety (DASS items) Maternal-foetal bonding (MAAS)	Rumination in 1st trimester is predictive of MAAS intensity scores in 3rd trimester (when controlling for depression, anxiety and demographic variables at time 1). Worry in 1st trimester is predictive of depression and anxiety in 3rd trimester (when controlling for depression, anxiety and demographic variables at time 1). Small effects: 0.8–3% variance.
*Experimental studies*			
Stein et al. (2012)	N = 253: (GAD n = 90, MDD n = 57; control n = 106) Infants 10 mths old. UK.	Randomised to either worry/rumination prime (WRP) or neutral prime (NP) and instructed to think about this topic for 5 min. Live interactions with infant observed and coded, before and after prime.	WRP resulted in more negative thoughts, higher thought recurrence and more self-focus relative to NP. Compared with controls, WRP had a more negative impact on maternal responsiveness to infant vocalization for GAD, and to a lesser extent for MDD; WRP also led to decreased maternal vocalization for GAD. Across the whole sample, WRP was associated with increased child vocalization relative to NP.
O'Mahen, Boyd, and Gashe (2015)	N = 59 dysphoric (BDI-II score ≥14); N = 39 non-dysphoric (BDI-II score <14). Infants aged 12mths or under. UK.	Randomly allocated to rumination or distraction condition – 8mins thought, guided by statements, e.g. rumination: “think about what your feelings might mean”, distraction: “think about a double-decker bus driving down a street”. Postnatal Parental Problem-Solving Task.	Dysphoric mothers in the rumination condition demonstrated poorer problem-solving effectiveness and less confidence in problem-solving compared to all other groups.

BADS = Behavioural Activation for Depression Scale; BDI = Beck Depression Inventory; DASS = Depression, Anxiety and Stress Scale; EPDS = Edinburgh Postnatal Depression Scale; IBQ-R = Infant Behaviour Questionnaire – Revised; MAAS = Maternal Antenatal Attachment Scale; MDQ = Major Depression Questionnaire; MIRI = Maternal Infant Responsiveness Instrument; PBQ = Postpartum Bonding Questionnaire; PBRS = Positive Beliefs about Rumination Scale; PSI = Parenting Stress Index; PSWQ-PW = Penn State Worry Questionnaire – Past Week; PTQ = Perseverative Thinking Questionnaire; RPA = Responses to Positive Affect questionnaire; RRS = Ruminative Response Scale; SASS = Social Adaptation Self-Evaluation Scale; SCID-IV = Structured Clinical Interview for DSM Disorders; SPS = Social Provisions Scale; STSS = Silencing the Self Scale.

## Footnotes

1 The majority of the research in this area has focused on mothers and so the mother-infant relationship is particularly emphasised throughout this review. However, we recognise that fathers are also involved in parenting, and similar processes are likely to apply. There is a clear need for additional research to examine this directly.

## References

[bib2] Barnum S., Woody M., Gibb B. (2013). Predicting changes in depressive symptoms from pregnancy to postpartum: the role of brooding rumination and negative inferential styles. Cognitive Therapy and Research.

[bib3] Barrett J., Fleming A.S. (2011). Annual research review: all mothers are not created equal: neural and psychobiological perspectives on mothering and the importance of individual differences. Journal of Child Psychology and Psychiatry.

[bib4] Beck C.T. (1992). The lived experience of postpartum depression: a phenomenological study. Nursing Research.

[bib5] Beck C.T. (2002). Postpartum depression: a metasynthesis. Qualitative Health Research.

[bib6] Bigelow A.E., MacLean B.K., MacDonald D. (1996). Infants' response to live and replay interactions with self and mother. Merrill-Palmer Quarterly.

[bib7] Bigelow A.E., Rochat P. (2006). Two-month-old infants' sensitivity to social contingency in mother-infant and stranger-infant interaction. Infancy.

[bib8] Bornstein M.H. (2014). Human infancy…and the rest of the lifespan. Annual Review of Psychology.

[bib9] Bornstein M.H., Manian N. (2013). Maternal responsiveness and sensitivity reconsidered: some is more. Development and Psychopathology.

[bib10] Bornstein M.H., Tamis-LeMonda C.S. (1989). Maternal responsiveness and cognitive development in children. New Directions in Child Development.

[bib11] Davis R., Nolen-Hoeksema S. (2000). Cognitive inflexibility among ruminators and nonruminators. Cognitive Therapy and Research.

[bib12] De Lissnyder E., Derakshan N., De Raedt R., Koster E.H. (2011). Depressive symptoms and cognitive control in a mixed antisaccade task: specific effects of depressive rumination. Cognition & Emotion.

[bib14] De Raedt R., Hertel P.T., Watkins E.R. (2015). Mechanisms of repetitive thinking: introduction to the special series. Clinical Psychological Science.

[bib15] Demeyer I., De Lissnyder E., Koster E.H., De Raedt R. (2012). Rumination mediates the relationship between impaired cognitive control for emotional information and depressive symptoms: a prospective study in remitted depressed adults. Behaviour Research and Therapy.

[bib16] Dimidjian S., Goodman S.H., Felder J.N., Gallop R., Brown A.P., Beck A. (2016). Staying well during pregnancy and the postpartum: a pilot randomized trial of mindfulness-based cognitive therapy for the prevention of depressive relapse/recurrence. Journal of Consulting and Clinical Psychology.

[bib17] Ehring T., Watkins E.R. (2008). Repetitive negative thinking as a transdiagnostic process. International Journal of Cognitive Therapy.

[bib18] Ehring T., Zetsche U., Weidacker K., Wahl K., Schonfeld S., Ehlers A. (2011). The perseverative thinking questionnaire (PTQ): validation of a content-independent measure of repetitive negative thinking. Journal of Behavior Therapy and Experimental Psychiatry.

[bib19] Everaert J., Koster E.H., Derakshan N. (2012). The combined cognitive bias hypothesis in depression. Clinical Psychology Review.

[bib20] Eysenck M.W., Derakshan N. (2011). New perspectives in attentional control theory. Personality and Individual Differences.

[bib21] Eysenck M.W., Derakshan N., Santos R., Calvo M.G. (2007). Anxiety and cognitive performance: attentional control theory. Emotion.

[bib22] Genet J.J., Siemer M. (2011). Flexible control in processing affective and non-affective material predicts individual differences in trait resilience. Cognition & Emotion.

[bib23] Ginsburg G.S., Grover R.L., Cord J.J., Ialongo N. (2006). Observational measures of parenting in anxious and nonanxious mothers: does type of task matter?. Journal of Clinical Child and Adolescent Psychology.

[bib25] Hayes S., Hirsch C., Mathews A. (2008). Restriction of working memory capacity during worry. Journal of Abnormal Psychology.

[bib26] Hayes S.C., Strosahl K.D., Wilson K.G. (2003). Acceptance and commitment therapy: An experiential approach to behavior change.

[bib27] Hertel P.T., Reisberg D., Hertel P. (2004). Memory for emotional and non-emotional events in depression: a question of habit. Memory and emotion.

[bib28] Hertel P.T., Mathews A. (2011). Cognitive bias modification: past perspectives, current findings, and future applications. Perspectives on Psychological Science.

[bib29] Hirsch C.R., Mathews A. (2012). A cognitive model of pathological worry. Behaviour Research and Therapy.

[bib30] Howard L.M., Molyneaux E., Dennis C.L., Rochat T., Stein A., Milgrom J. (2014). Non-psychotic mental disorders in the perinatal period. Lancet.

[bib31] Isa Alfaraj A.M.A., Spada M.M., Nikčević A.V., Puffett A., Meer S. (2009). Positive beliefs about rumination in depressed and non-depressed pregnant women: a preliminary investigation. Journal of Reproductive and Infant Psychology.

[bib32] Jones N.P., Siegle G.J., Thase M.E. (2008). Effects of rumination and initial severity on remission to cognitive therapy for depression. Cognitive Therapy and Research.

[bib33] Joormann J. (2006). Differential effects of rumination and dysphoria on the inhibition of irrelevant emotional material: evidence from a negative priming task. Cognitive Therapy and Research.

[bib36] Keller H., Lohaus A., Völker S., Cappenberg M., Chasiotis A. (1999). Temporal contingency as an independent component of parenting behavior. Child Development.

[bib37] Kirkanski K., Thompson R.J., Sorenson J.E., Sherdell L., Gotlib I.H. (2015). Rumination and worry in daily life: examining the naturalistic validity of theoretical constructs. Clinical Psychological Science.

[bib88] Martell C.R., Dimidjian S., Herman-Dunn R. (2012). Behavioral activation for depression: A clinician's guide.

[bib39] Mathews A., MacLeod C. (2005). Cognitive vulnerability to emotional disorders. Annual Review of Clinical Psychology.

[bib40] Mesman J., van Ijzendoorn M.H., Bakermans-Kranenburg M.J. (2009). The many faces of the still-face paradigm: a review and meta-analysis. Developmental Review.

[bib41] Messinis L., Vlahou C.H., Tsapanos V., Tsapanos A., Spilioti D., Papathanasopoulos P. (2010). Neuropsychological functioning in postpartum depressed versus nondepressed females and non postpartum controls. Journal of Clinical and Experimental Neuropsychology.

[bib42] Michalak J., Hölz A., Teismann T. (2011). Rumination as a predictor of relapse in mindfulness-based cognitive therapy for depression. Psychology and Psychotherapy: Theory, Research and Practice.

[bib43] Miyake A., Friedman N.P., Emerson M.J., Witzki A.H., Howerter A., Wager T.D. (2000). The unity and diversity of executive functions and their contributions to complex “Frontal Lobe” tasks: a latent variable analysis. Cognitive Psychology.

[bib44] Moher D., Liberati A., Tetzlaff J., Altman D.G., PRISMA group (2009). Preferred reporting items for systematic reviews and meta-analyses: the PRISMA statement. PLoS Medicine.

[bib45] Mor N., Daches S. (2015). Ruminative thinking: lessons learned from cognitive training. Clinical Psychological Science.

[bib46] Müller D., Teismann T., Havemann B., Michalak J., Seehagen S. (2013). Ruminative thinking as a predictor of perceived postpartum mother–infant bonding. Cognitive Therapy and Research.

[bib47] Murray L., Halligan S.L., Cooper P., Bremner J.G., Wachs T.D. (2010). Effects of postnatal depression on mother-infant interactions and child development. Handbook of infant development (2nd ed.).

[bib48] Nadel J., Carchon I., Kervella C., Marcelli D., Réserbat-Plantey D. (2001). Expectancies for social contingency in 2-month-olds. Developmental Science.

[bib49] Nolen-Hoeksema S., Wisco B.E., Lyubomirsky S. (2008). Rethinking rumination. Perspectives on Psychological Science.

[bib50] O'Mahen H.A., Boyd A., Gashe C. (2015). Rumination decreases parental problem-solving effectiveness in dysphoric postnatal mothers. Journal of Behavior Therapy and Experimental Psychiatry.

[bib51] O'Mahen H.A., Flynn H.A., Nolen-Hoeksema S. (2010). Rumination and interpersonal functioning in perinatal depression. Journal of Social and Clinical Psychology.

[bib52] O'Mahen H.A., Karl A., Moberly N., Fedock G. (2015). The association between childhood maltreatment and emotion regulation: two different mechanisms contributing to depression?. Journal of Affective Disorders.

[bib53] Ochsner K.N., Gross J.J. (2005). The cognitive control of emotion. Trends in Cognitive Sciences.

[bib54] Papageorgiou C., Wells A. (2001). Metacognitive beliefs about rumination in recurrent major depression. Cognitive and Behavioral Practice.

[bib55] Papageorgiou C., Wells A. (2003). An empirical test of a clinical metacognitive model of rumination and depression. Cognitive Therapy and Research.

[bib56] Philippot P., Brutoux F. (2008). Induced rumination dampens executive processes in dysphoric young adults. Journal of Behavior Therapy and Experimental Psychiatry.

[bib57] Pio de Almeida L.S., Jansen K., Köhler C.A., Pinheiro R.T., da Silva R.A. (2012). Working and short-term memories are impaired in postpartum depression. Journal of Affective Disorders.

[bib58] Poobalan A.S., Aucott L.S., Ross L., Smith W.C., Helms P.J., Williams J.H. (2007). Effects of treating postnatal depression on mother-infant interaction and child development: systematic review. British Journal of Psychiatry.

[bib59] Porter R.J., Bowie C.R., Jordan J., Malhi G.S. (2013). Cognitive remediation as a treatment for major depression: a rationale, review of evidence and recommendations for future research. Australia and New Zealand Journal of Psychiatry.

[bib60] Psychogiou L., Parry E. (2014). Why do depressed individuals have difficulties in their parenting role?. Psychological Medicine.

[bib61] Raes F., Smets J., Wessel I., Van Den Eede F., Nelis S., Franck E., Hanssens M. (2014). Turning the pink cloud grey: dampening of positive affect predicts postpartum depressive symptoms. Journal of Psychosomatic Research.

[bib62] Schmaling K.B., Dimidjian S., Katon W., Sullivan M. (2002). Response styles among patients with minor depression and dysthymia in primary care. Journal of Abnormal Psychology.

[bib63] Schmidt D., Seehagen S., Vocks S., Schneider S., Teismann T. (2016). Predictive importance of antenatal depressive rumination and worrying for maternal-foetal attachment and maternal well-being. Cognitive Therapy and Research.

[bib64] Segal Z.V., Williams J.M.G., Teasdale J.D. (2013). Mindfulness-based cognitive therapy for depression: A new approach to preventing relapse.

[bib65] Shin H., Park Y.J., Kim M.J. (2006). Predictors of maternal sensitivity during the early postartum period. Journal of Advanced Nursing.

[bib66] Siddiqui A., Hägglöf B. (2000). Does maternal prenatal attachment predict postnatal mother-infant interaction?. Early Human Development.

[bib68] Stanley C., Murray L., Stein A. (2004). The effect of postnatal depression on mother-infant interaction, infant response to the still-face perturbation, and performance on an instrumental learning task. Developement and Psychopathology.

[bib69] Stefanopoulou E., Hirsch C.R., Hayes S., Adlam A., Coker S. (2014). Are attentional control resources reduced by worry in generalized anxiety disorder?. Journal of Abnormal Psychology.

[bib70] Stein A., Craske M.G., Lehtonen A., Harvey A., Savage-McGlynn E., Davies B., Counsell N. (2012). Maternal cognitions and mother-infant interaction in postnatal depression and generalized anxiety disorder. Journal of Abnormal Psychology.

[bib71] Stein A., Lehtonen A., Harvey A.G., Nicol-Harper R., Craske M. (2009). The influence of postnatal psychiatric disorder on child development. Is maternal preoccupation one of the key underlying processes?. Psychopathology.

[bib72] Stein A., Pearson R.M., Goodman S.H., Rapa E., Rahman A., McCallum M., Pariante C.M. (2014). Effects of perinatal mental disorders on the fetus and child. Lancet.

[bib73] Striano T., Henning A., Stahl D. (2006). Sensitivity to interpersonal timing at 3 and 6 months of age. Interaction studies.

[bib74] Tester-Jones M., O'Mahen H., Watkins E., Karl A. (2015). The impact of maternal characteristics, infant temperament and contextual factors on maternal responsiveness to infant. Infant Behavior and Development.

[bib75] Topper M., Emmelkamp P.M.G., Ehring T. (2010). Improving prevention of depression and anxiety disorders: repetitive negative thinking as a promising target. Applied & Preventive Psychology.

[bib76] Treynor W., Gonzalez R., Nolen-Hoeksema S. (2003). Rumination reconsidered: a psychometric analysis. Cognitive Therapy and Research.

[bib77] Tronick E., Als H., Adamson L., Wise S., Brazelton T.B. (1978). The infant's response to entrapment between contradictory messages in face-to-face interaction. Journal of the American Academy of Child Psychiatry.

[bib79] Watkins E., Brown R.G. (2002). Rumination and executive function in depression: an experimental study. Journal of Neurology Neurosurgery & Psychiatry.

[bib80] Watkins E.R., Nolen-Hoeksema S. (2014). A habit-goal framework of depressive rumination. Journal of Abnormal Psychology.

[bib81] Webb R., Ayers S. (2014). Cognitive biases in processing infant emotion by women with depression, anxiety and post-traumatic stress disorder in pregnancy or after birth: a systematic review. Cognition & Emotion.

[bib82] Wells A. (2009). Metacognitive therapy for anxiety and depression.

[bib85] Whitmer A.J., Banich M.T. (2007). Inhibition versus switching deficits in different forms of rumination. Psychological Science.

[bib86] Whitmer A.J., Gotlib I.H. (2013). An attentional scope model of rumination. Psychological Bulletin.

[bib87] Williamson J.A., O'Hara M.W., Stuart S., Hart K.J., Watson D. (2015). Assessment of postpartum depressive symptoms: the importance of somatic symptoms and irritability. Assessment.

